# Achieving Acetylcholine Receptor Clustering in Tissue-Engineered Skeletal Muscle Constructs *In vitro* through a Materials-Directed Agrin Delivery Approach

**DOI:** 10.3389/fphar.2016.00508

**Published:** 2017-01-11

**Authors:** John B. Scott, Catherine L. Ward, Benjamin T. Corona, Michael R. Deschenes, Benjamin S. Harrison, Justin M. Saul, George J. Christ

**Affiliations:** ^1^Wake Forest Institute for Regenerative Medicine, Wake Forest University Health Sciences, Winston-SalemNC, USA; ^2^Virginia Tech – Wake Forest University School of Biomedical Engineering and Sciences, Wake Forest University Biomedical Engineering, Winston-SalemNC, USA; ^3^US Army Institute for Surgical Research, San AntonioTX, USA; ^4^Department of Neuroscience, College of William and Mary, WilliamsburgVA, USA; ^5^Department of Chemical, Paper and Biomedical Engineering, Miami University, OxfordOH, USA; ^6^Department of Biomedical Engineering and Department of Orthopaedic Surgery, University of Virginia, CharlottesvilleVA, USA

**Keywords:** bioreactor, drug delivery, fibrin, microspheres, tissue engineering, regenerative medicine

## Abstract

Volumetric muscle loss (VML) can result from trauma, infection, congenital anomalies, or surgery, and produce permanent functional and cosmetic deficits. There are no effective treatment options for VML injuries, and recent advances toward development of muscle constructs lack the ability to achieve innervation necessary for long-term function. We sought to develop a proof-of-concept biomaterial construct that could achieve acetylcholine receptor (AChR) clustering on muscle-derived cells (MDCs) *in vitro*. The approach consisted of the presentation of neural (Z+) agrin from the surface of microspheres embedded with a fibrin hydrogel to muscle cells (C2C12 cell line or primary rat MDCs). AChR clustering was spatially restricted to areas of cell (C2C12)-microsphere contact when the microspheres were delivered in suspension or when they were incorporated into a thin (2D) fibrin hydrogel. AChR clusters were observed from 16 to 72 h after treatment when Z+ agrin was adsorbed to the microspheres, and for greater than 120 h when agrin was covalently coupled to the microspheres. Little to no AChR clustering was observed when agrin-coated microspheres were delivered from specially designed 3D fibrin constructs. However, cyclic stretch in combination with agrin-presenting microspheres led to dramatic enhancement of AChR clustering in cells cultured on these 3D fibrin constructs, suggesting a synergistic effect between mechanical strain and agrin stimulation of AChR clustering *in vitro*. These studies highlight a strategy for maintaining a physiological phenotype characterized by motor endplates of muscle cells used in tissue engineering strategies for muscle regeneration. As such, these observations may provide an important first step toward improving function of tissue-engineered constructs for treatment of VML injuries.

## Introduction

The ability to voluntarily control skeletal muscle is critically important to key daily functions such as locomotion, breathing, and postural support. Skeletal muscle possesses an inherent capacity for self-repair following injury. The repair process consists of a series of phases that start with inflammation followed by satellite cell mobilization and, ultimately, to maturation of myofibers and remodeling of muscle architecture (Charge and Rudnicki, 2004; Jarvinen et al., 2005; Ambrosio et al., 2009; Ciciliot and Schiaffino, 2010). However, this repair process is insufficient in more extensive injuries, resulting in extensive scar tissue formation (Jarvinen et al., 2005). These larger injuries in which the muscle damage results in permanent functional and cosmetic defects are referred to as VML injuries (Grogan and Hsu, 2011). Although VML can result from a variety of causes including congenital abnormalities, acquired disease and tumor resection, the most devastating injuries generally result from trauma. Traumatic VML injuries in the civilian population result from causes such as automobile accidents (and gun shot wounds) (Mazurek and Ficke, 2006) and in military personnel from high impact forces (Mazurek and Ficke, 2006; Owens et al., 2008; Lew et al., 2010).

Surgical treatment of VML injuries by autologous tissue transfer is limited by limited donor tissue, donor site morbidity and poor engraftment (Khouri et al., 1998; Lin et al., 1999; Lawson and Levin, 2007). Alternative strategies for VML repair include cell transplantation (Ni et al., 2014; Oshima et al., 2014) platelet-rich plasma (Hamid et al., 2014), growth factors (Dumont and Frenette, 2013), hyperbaric oxygen inhalation (Horie et al., 2014), gene therapy (Jang and Shea, 2006), and traditional pharmacological approaches (Vignaud et al., 2005; Kobayashi et al., 2013). However, none of these strategies restore satisfactory function.

Because existing clinical treatments are lacking, numerous tissue engineering approaches are being investigated as alternatives to achieve skeletal muscle repair and regeneration. The tissue engineering triad consists of some combination of biomaterial scaffolds, cells, and signals (e.g., mechanical or chemical signaling) (Lanza et al., 2014), and each of these have been investigated for capability to achieve skeletal muscle regeneration. Synthetic or naturally derived biomaterial scaffolds have been used alone, without addition of cells or signals (Badylak et al., 2001; Crow et al., 2007; Kin et al., 2007; Dufrane et al., 2008; Merritt et al., 2010b; Valentin et al., 2010; McKeon-Fischer et al., 2011; Perniconi et al., 2011; Sicari et al., 2012; Turner et al., 2012; Sun et al., 2013). Alternatively, biomaterials have been used in conjunction with cells in an effort to further improve *in vivo* outcomes (Fujita et al., 2010; Borselli et al., 2011; Hinds et al., 2011; Machingal et al., 2011; Page et al., 2011; Rossi et al., 2011; Corona et al., 2012, 2013, 2014; Monge et al., 2012; Du et al., 2013; Martin et al., 2013; Williams et al., 2013; Juhas and Bursac, 2014; Juhas et al., 2014; VanDusen et al., 2014; Wang et al., 2014) (for a recent review of these studies, see, Christ et al., 2015). Incorporation of signals has included chemical cues such as pharmacological molecules/growth factors (Falco et al., 2011; Sato et al., 2011; Bian and Bursac, 2012; Koning et al., 2012; Wang et al., 2012, 2014; Yun et al., 2012; Ko et al., 2013) or mechanical cues such as mechanical stretch (Vandenburgh and Karlisch, 1989; Vandenburgh et al., 1989, 1991; Moon du et al., 2008; Machingal et al., 2011; Corona et al., 2012). In each case, the goal is to identify a strategy in which a micro-environment more favorable to skeletal muscle regeneration can be achieved.

Despite progress toward improved outcomes (e.g., contractile force), we are not aware of any reports of full functional recovery in biologically relevant animal models. One possible reason for the lack of long-term success is the micro-environmental role played by axons that innervate the muscle and form NMJs (Sanes and Lichtman, 2001; Madhavan and Peng, 2005; Witzemann, 2006; Wu et al., 2010). In native, healthy muscle tissue innervation is indicated by the formation of a mature post-synaptic apparatus known as the motor end plate (MEP) (Sanes and Lichtman, 2001; Madhavan and Peng, 2005; Witzemann, 2006; Wu et al., 2010). In non-VML injuries to skeletal muscle, re-innervation is achieved when endplates remain present (Frank et al., 1975; Carlson and Faulkner, 1988; Dedkov et al., 2002). Further, it is well-known that muscle cells atrophy if chronically denervated (Jarvinen et al., 2005). Thus, the lack of MEP in muscle cells used for current tissue engineering strategies for VML injuries, coupled to the slow rate of axon growth (∼1 mm/day) (Gutmann et al., 1942; Gordon et al., 2008; Kang and Lichtman, 2013) is likely a significant contributing factor to the lack of functional recovery in VML injury models.

Strategies capable of maintaining the presence of MEPs on tissue engineered skeletal muscle during the neural re-innervation process could have important implications on the ability to ultimately achieve functional restoration *in vivo*. In innervated skeletal muscle, the MEP is a complex structure in the muscle membrane that contains sodium–potassium ion channels, binding sites for acetylcholinesterase (the enzyme responsible for degradation of acetylcholine), and AChR clusters. Each of these components is critical to the function of the MEP and, ultimately, the function of a neuro-muscular junction. Thus, an important step toward the formation of MEPs is the formation of AChR clusters (Daggett et al., 1996; Burgess et al., 1999; Sanes and Lichtman, 2001; Bezakova and Ruegg, 2003; Madhavan and Peng, 2005; Witzemann, 2006; Wu et al., 2010).

**FIGURE 1:**
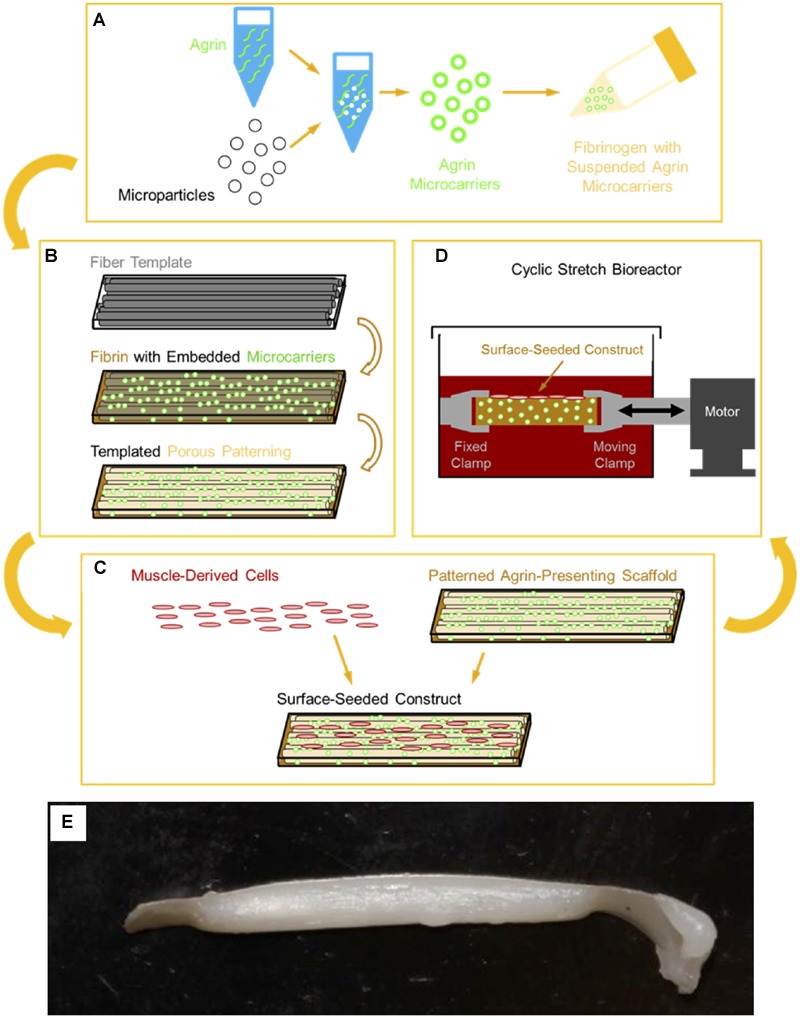
**Schematic of 3D tissue engineered muscle construct fabrication, seeding, and preconditioning. (A)** Pharmacologic delivery vehicles were created by covalent immobilization of agrin on a microsphere surface. These agrin microspheres were then suspended in a solution of fibrinogen, which was **(B)** polymerized to fibrin around a sacrificial template of polymer fibers, as previously described (Scott et al., 2011). Selective dissolution of the template resulted in a patterned hydrogel material of known mechanical properties (Scott et al., 2011) that presented agrin via embedded microspheres. **(C)** Scaffolds were then seeded by manually pipetting a suspension of MDCs over the scaffold surface. Resident cells then received a combination of pharmacologic cues from scaffold agrin and/or **(D)** mechanical cues from cyclic stretch in a bioreactor. **(E)** A tissue engineered construct fabricated using this approach after cell seeding and bioreactor preconditioning.

However, we are unaware of any biomaterials approaches compatible with tissue engineering strategies that have investigated such an approach. In this report, we describe our initial efforts to develop biomaterial constructs capable of inducing AChR clustering as a step toward generation of MEPs that ultimately may promote better *in vivo* function of tissue engineered skeletal muscle for VML. The approach described in this report consisted of adsorbing or covalently coupling neural agrin (a known AChR clustering molecule) onto microspheres capable of achieving a spatially regulated delivery of agrin (see next paragraph) to cells in order to promote AChR clustering. Further, these microspheres were then incorporated into fibrin hydrogels, as shown schematically in **Figure 1**, in order to allow presentation of the agrin from the fibrin hydrogels in a localized manner that can achieve localized AChR clustering. Further, the subsequent immobilization of agrin-bound microparticles in the hydrogels is expected to greatly reduce diffusion of the agrin away from the construct, potentially enhancing its long-term activity.

Neural agrin is a heparan sulfate proteoglycan noted for its ability to induce clustering of plasma membrane-bound AChRs in skeletal muscle cells. This clustering behavior is unique to agrin isoforms featuring a splice insert at the Z-site that is specific to nerve-derived (Z+) agrin (Burgess et al., 1999). Studies to date exploring neural agrin functionalization for skeletal muscle tissue engineering applications have shown enhanced AChR cluster formation, dystrophin gene expression, and force production in tissue engineered skeletal muscle constructs when cultured in media containing agrin (Bian and Bursac, 2012; Ko et al., 2013). However, these studies have exclusively employed soluble agrin conditioning *in vitro*, which would not be capable of maintaining the localized agrin signal required within the TEMR construct once implanted *in vivo*. This distinction is important, as neural agrin *in vivo* is immobilized in the synaptic basal lamina, and thus, restricted to areas of nerve-muscle contact (Wu et al., 2010).

Fibrin hydrogels were used as the biomaterial for these studies because their hydrogel nature is highly compatible with incorporation of agrin-coated microspheres in a way not as readily achieved with other biomaterials such as bladder acellular matrix. These materials are also compatible with strategies to achieve alignment of myofibers and, importantly they provide sufficient mechanical integrity for *in vitro* bioreactor pre-conditioning when fabricated by a sacrificial templating method (Scott et al., 2011). This material requirement is important, as previous studies by our group have shown that within 2 months of implantation, TEMR constructs subjected to biomechanical preconditioning *in vitro* produce significant improvements in the rate and/or magnitude of functional recovery compared to implantation of the material alone or the TEMR constructs without bioreactor preconditioning (Machingal et al., 2011; Corona et al., 2012). These findings clearly indicated that *in vitro* preconditioning of TEMR constructs has an important *impact* on subsequent functional recovery of VML injuries following implantation *in vivo.*

The overarching hypothesis underlying the studies in this manuscript is that one reason for lack of functional restoration in tissue-engineered approaches to VML is the lack of innervation from the native peripheral system, which itself is related to lack of AChR clustering. The goals of these studies, then, were three-fold. First, we sought to describe the development of a novel approach to incorporate microsphere-immobilized neural agrin into biomaterial constructs. We then investigated the enhancement of AChR clusters on either a myoblast cell line (C2C12 cells) or on primary rat MDCs in order to assess the efficacy of the strategy for TEMR applications. Lastly, we sought to investigate whether the microsphere agrin-delivery strategy would be compatible and/or synergistic with our previous efforts to apply cyclic stretch to TEMR constructs. While these studies are largely proof-of-concept in nature, the results demonstrate an important step in developing biomimetic materials that achieve and maintain a key phenotypic characteristic of cells to be used for TEMR approaches to VML. Incorporation of a strategy such as the one described here may play a key role in the further development of tissue-engineered scaffolds for VML.

## Materials and Methods

### Preparation of Microspheres with Adsorbed or Covalently Coupled Agrin

For control experiments in which agrin was delivered in solution-phase (not adsorbed to microspheres), culture medium was prepared by diluting recombinant rat agrin (R&D Systems, Minneapolis, MN, USA) stock at 100 μg/mL (∼1.11 μM) in sterile PBS by using an appropriate culture medium (specific to each experiment described below). The final agrin concentration was 50 ng/mL (∼0.56 nM).

Agrin-adsorbed (non-covalently bound) microspheres were prepared from either 10 μm diameter Polybead^®^ microspheres for epifluorescence experiments or 10 μm diameter Fluoresbrite^®^ yellow green microspheres for confocal experiments described below (both Polysciences, Inc., Warrington, PA, USA). Different beads were used for these experiments to optimize fluorescence on each system based on filters/lasers available. Microspheres were first centrifuged from stock into a pellet and then sterilized by resuspension to a concentration of ∼34 million particles/mL in 95% v/v ethanol in water and agitation on a lab roller for at least 15 min at ∼22°C. Subsequent steps were carried out in a biological safety cabinet to maintain sterility. Microspheres were centrifuged and washed in sterile water twice to remove residual ethanol before being resuspended from a pellet to a concentration of ∼84 million particles/mL (calculated by dilution over sequential steps of the listed stock concentration provided by the manufacturer) in 100 μg/mL agrin stock and agitated on a lab roller for at least 1 h at ∼22°C to allow for agrin adsorption. Finally, microspheres were centrifuged and washed in sterile water three times to remove non-adsorbed agrin and then resuspended to a functional microsphere stock concentration of ∼48 million particles/mL in sterile water, in which form they were stored statically at 4°C for up to 2 months before use.

Covalently bound agrin microspheres were prepared by using the zero-length crosslinker *N*-(3-dimethylaminopropyl)-*N*′-ethylcarbodiimide hydrochloride (EDC, Sigma-Aldrich, St. Louis, MO, USA) to link amine groups on agrin molecules to either 10 μm diameter Fluoresbrite^®^ yellow green carboxylate microspheres (Polysciences) or 10 μm diameter SiO_2_ microspheres (Corpuscular, Cold Spring, NY, USA) depending on the experiment described below. Fluoresbrite^®^ polystyrene microspheres were selected because they provided better fluorescence for confocal microscopy. SiO_2_ microsphere were used in some experiments because the other microspheres were not compatible with the acetone used for fabrication of 3-D fibrin hydrogels formed by a sacrificial templating approach (see below). Though SiO_2_ microspheres were translucent under confocal microscopy, they were visible within the scaffold as they contrasted with the surrounding autofluorescent fibrin. Each type of microsphere was first centrifuged from stock into a pellet and then washed twice in MES buffer (pH 5.7), consisting of 0.1 M MES (Acros Organics, Geel, Belgium) and 0.5 M NaCl (Sigma). Microspheres were then centrifuged and resuspended to ∼42 million particles/mL in an intermediate crosslinking solution consisting of MES buffer with 10 mg/mL (52 mM) EDC and 10 mg/mL (46 mM) sulfo-NHS (Sigma). This intermediate suspension was agitated on a lab roller for 15 min at ∼22°C before being quickly centrifuged and washed twice in PBS (Thermo Fisher Scientific), pH 7.4. These functionalized microspheres were centrifuged to a pellet and crosslinking to agrin was accomplished by resuspending to a concentration of ∼42 million particles/mL in a 50 μg/mL (∼560 nM) solution of agrin in PBS and agitating on a lab roller for at least 6 h at ∼22°C. Agrin-bound microspheres were subsequently centrifuged, resuspended at a concentration of ∼34 million particles/mL in a solution of 95% v/v ethanol in water, and agitated on a lab roller for at least 30 min at ∼22°C to both remove any agrin simply adsorbed to the microsphere surface as well as to sterilize the microspheres. Finally, microspheres were centrifuged and washed twice with sterile water in a biological safety cabinet to remove residual ethanol and then resuspended to a functional microspheres stock concentration of ∼48 million particles/mL in sterile water, in which form they were stored statically at 4°C for up to 3 months before use.

Several negative controls were used as described in experiments below. As a negative control to agrin-adsorbed microspheres, 10 μm diameter Polybead^®^ microspheres were sterilized in ethanol and washed in sterile water as above, but were not subjected to further manipulation. As a negative control to covalently bound agrin microspheres in 2D model systems, Polybead^®^ carboxylate microspheres or Fluoresbrite^®^ yellow green carboxylate microspheres were sterilized in ethanol and washed in sterile water as above, but were not subjected to further manipulation. Covalently bound BSA (Sigma) was used as another negative control for the covalently bound agrin microspheres. The BSA was covalently attached to Polybead^®^ carboxylate microspheres as described above for agrin except that BSA at 36.7 μg/mL (∼560 nM) in 50% v/v PBS/deionized water was used.

### Culture Medium Formulation

**FIGURE 2:**
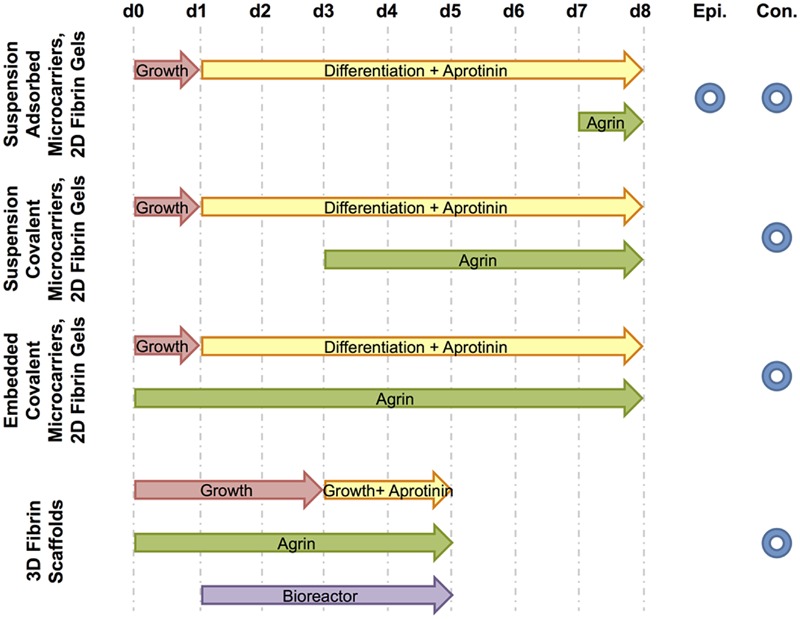
**Schematic of treatments and fluorescence microscopy techniques used.** For each experiment, culture media and agrin treatment time courses are shown from the day of cell seeding (d0) to the end of the experiment (d8 for 2D experiments or d5 for the 3D experiment). The period of bioreactor preconditioning is also shown for the 3D experiment. Further, each experiment is designated by the type of fluorescence microscopy used to image relevant samples – either epifluorescence (Epi.) and / or confocal (Con.).

Four different culture media formulations were used in experiments described below, with all components from Thermo Fisher Scientific (Waltham, MA, USA) unless otherwise specified. C2C12 growth medium consisted of high-glucose Dulbecco’s modified Eagle’s medium (DMEM), 10% v/v heat-inactivated fetal bovine serum (FBS), and 1% penicillin/streptomycin. Differentiation medium, myogenic medium, and rMDC growth medium (previously referred to as seeding medium) compositions were prepared as previously described (Machingal et al., 2011; Corona et al., 2012). Experimental cell cultures *in vitro* described below were supplemented with agrin either dissolved within culture medium or bound to the surface of microspheres by adsorption or covalent crosslinking. **Figure 2** schematically summarizes experimental treatments and the time course over which each was applied. Control microspheres (no agrin or with BSA) were prepared as described above. As a negative control to solution-phase agrin in culture medium, culture medium of the appropriate type was used without added agrin.

### C2C12 Cell Response to Solution-Phase Agrin and Agrin-Adsorbed Microspheres on 2D Fibrin Gels *In vitro*

In order to assess whether agrin adsorbed to microspheres could achieve similar AChR clustering to solution-phase agrin, we delivered agrin alone or adsorbed to microspheres from solution or in suspension, respectively. These solutions were provided to C2C12 cells seeded on a thin sheet of fibrin on a glass coverslip, which we refer to as a 2-D gel. Both sides of 12 mm diameter round glass coverslips (Leica Microsystems, Buffalo Grove, IL, USA) were mechanically roughened with sandpaper to provide an adherent surface for the fibrin hydrogels. We note that without roughening, we were unable to achieve stable contact between fibrin hydrogels and the glass coverslips, but we did not observe any optical interference and all samples were treated in the same fashion. The coverslips were then rinsed with DI water, then dried. 7 μL fibrinogen (Sigma) at 10 mg/mL (∼29 μM) in PBS was mixed with 10 μL of a thrombin working solution containing 16.7 U/mL thrombin and 367 μg/mL (∼3.3 mM) CaCl_2_ (both Sigma) in PBS and spreading the mixture evenly across the glass surface. After gelation, coverslips were placed into individual wells of a 24-well dish and then sterilized by exposure to ≥1 MRad γ radiation.

C2C12 mouse myoblasts (ATCC, Manassas, VA, USA) were suspended in C2C12 growth medium to a concentration of 50,000 cells/mL and then 1 mL of this suspension was added to each well-containing a fibrin-coated coverslip. After 16–24 h for cell attachment, growth medium was removed and replaced with differentiation medium supplemented with aprotinin (Sigma) at 20 μg/ml (∼3.1 μM) to inhibit fibrinolysis. This supplemented differentiation medium was exchanged every 2–3 days. After 6 days of culture in differentiation medium, either (1) aprotinin-supplemented differentiation medium alone, (2) a solution of agrin in aprotinin-supplemented differentiation medium, (3) a suspension of control microspheres (no agrin) at ∼1.2 million particles/mL in aprotinin-supplemented differentiation medium, or (4) a suspension of agrin-adsorbed microspheres at ∼1.2 million particles/mL in aprotinin-supplemented differentiation medium was added to each well. Cells were then left for an additional 16–24 h to allow any AChR clustering to occur before being evaluated by histology and epifluorescence microscopy as described below. Concentrations of microspheres in suspension within culture medium here and in subsequent experiments were based on pilot studies that allowed enough microspheres to contact cells and remain throughout subsequent processing while not obscuring the cell layer during histological processing.

### C2C12 Cell Response to Covalently Bound Agrin Microspheres on 2D Fibrin Gels *In vitro*

After evaluation of the microspheres delivered from solution, we then investigated incorporation of the microspheres directly into 2D fibrin hydrogels to determine if AChR clustering could be obtained. Pilot studies indicated desorption of agrin from the microspheres during this process, so we elected to use covalently coupled agrin for these experiments. Aprotinin supplementation, medium changes, and histology were performed as above, but experimental particles presenting covalently immobilized agrin or control microspheres were added to C2C12 cell-seeded fibrin gels after only 2 days of culture in differentiation medium, allowing for 5 days of cell-microsphere contact. Staining, evaluation of AChR localization using confocal microscopy, and image processing were performed as described below.

Fibrin gels were polymerized on glass coverslips by methods similar to those above with a minor change that all materials were filter sterilized rather than subjected to gamma irradiation in order to avoid possible degradation of agrin. 10 μL of 10 mg/mL fibrinogen solution containing a suspension of ∼124 million/mL covalently bound agrin Fluoresbrite^®^ particles or of untreated control Fluoresbrite^®^ microspheres was polymerized by adding 2 μL of a solution consisting of 125 U/mL thrombin and 2.75 μg/mL (∼25 mM) CaCl_2_ in PBS. This concentration of microspheres was used to approximate a fibrin surface with a roughly equal number of microspheres to those provided in suspension in previous experiments. C2C12 cells were then seeded, cultured, stained, and imaged as above. Because microspheres were immobilized within the gel prior to cell seeding, no microspheres of any kind were added from the solution phase.

### 3D *In vitro* Model System for Evaluation of the Response of Rat MDCs to Agrin-Containing Fibrin Materials Combined with Mechanical Strain

We fabricated custom 3D fibrin hydrogels containing agrin microspheres in order to assess the behavior of cells within a construct suitable for cyclic strain bioreactor conditioning previously used by our group (see **Figure 1** for schematic overview). These 3D fibrin scaffolds were fabricated by methods similar to those previously described (Scott et al., 2011). CA fibers (generous donation of Eastman Chemical Company, Kingsport, TN, USA) ∼12 μm in diameter were arranged into an aligned mat roughly 35 mm long × 1 mm thick. Experimental covalently bound SiO_2_ agrin microspheres or control covalently bound BSA microspheres were suspended in 200 mg/mL fibrinogen at a density of ∼24 million particles/mL, and this suspension was added to the fibers until the mat was completely impregnated. Fibrinogen was polymerized to fibrin by adding a solution of 125 U/mL thrombin and 2.75 μg/mL (∼25 mM) CaCl_2_ in PBS and leaving undisturbed for at least 4 h at ∼22°C. The scaffold was then trimmed into individual smaller scaffolds measuring roughly 30 mm long × 3 mm wide × 1 mm thick using a razor blade. These were then sequentially washed, first in acetone and then in sterile PBS, to yield sterilized 3D scaffolds of known mechanical properties and morphology (Scott et al., 2011).

Separately, rat MDCs were obtained from 4 to 6 week old male Lewis rats (Charles River, Raleigh, NC, USA) and were isolated as previously described (Machingal et al., 2011; Corona et al., 2012). This isolation protocol was approved by the Wake Forest University Institutional Animal Care and Use Committee. Expanded rat MDCs were used for seeding at the second passage. Representative images of these cells are available as published previously (Machingal et al., 2011; Corona et al., 2012, 2014).

One 30 mm long × 3 mm wide piece of fibrin scaffold was then seeded with rat MDCs by manually pipetting ∼30 μL suspension per scaffold of 3 × 10^7^ cells/mL in rat MDC growth medium over a closely packed side-by-side arrangement of scaffolds. After 30 min for cell attachment, 700 μL of rat MDC growth medium per scaffold was added to immerse the cell-seeded scaffolds. The experimental groups were fibrin scaffolds with agrin microspheres or BSA microspheres (control). One group of each experimental cell-seeded scaffold were left mechanically undisturbed for 6 days (static culture), while a second group was removed from static culture after 16–24 h (to allow cell attachment) and then immediately clamped into the mounts of a custom-fabricated bioreactor. This bioreactor was similar to those previously described (Moon du et al., 2008; Machingal et al., 2011; Corona et al., 2012), consisting of a medium reservoir, a computer-controlled electromagnetic linear actuator, and a series of opposing clamps to suspend scaffolds within the medium reservoir while anchoring one scaffold end to a fixed point and the other to the linear actuator. The bioreactor was used to “precondition” cell-seeded scaffolds by subjecting them to 10% mechanical strain parallel to the long axis of scaffolds three times per minute for the first 5 min of every hour over a total time of 5 days, consistent with our previous reports (Moon du et al., 2008; Machingal et al., 2011; Corona et al., 2012).

In all groups, rat MDC growth medium was exchanged every 2–3 days and was supplemented with aprotinin starting on day 3. After 6 days, AChR localization in muscle cells associated with the scaffold was evaluated by histology and imaging methods described below.

### Histofluorescence

C2C12 cells on 2D fibrin gels were evaluated histologically while still attached to coverslips. Gel-adherent cells were fixed in 10% neutral-buffered formalin (Leica) for 3–5 min, washed three times for 5 min each in TBST (Dako, Glostrup, Denmark), blocked against non-specific binding by using a blocking buffer consisting of 5% v/v horse serum in TBST for at least 30 min at ∼22°C, stained for AChR localization by α-bungarotoxin conjugated with Alexa Fluor 594 (α-BTX, Invitrogen) at 100 nM dilution in blocking buffer for at least 1 h at ∼22°C in the dark, washed a further three times in TBST, mounted to glass slides with Vectashield containing DAPI (Vector Laboratories, Burlingame, CA, USA) and dried overnight at 4°C.

3D cell-seeded scaffolds were removed whole from bioreactors, fixed by immersion in 4% paraformaldehyde in PBS for 16–24 h at 4°C, and paraffin processed in an ASP300 tissue processor (Leica). Processed samples were then mounted into paraffin blocks and longitudinally sectioned on a microtome to 15 μm thickness, with the plane of the section perpendicular to the 30 mm × 3 mm plane of the scaffold. Tissue sections were immobilized on glass slides, dried for 16–48 h at 60°C, and then left indefinitely at ∼22°C until stained. Prior to staining, slides were deparaffinized by sequential washes in xylenes, ethanol, and tap water. Staining was then performed as for 2D samples, beginning with the initial three washes of TBST. We note that no antigen retrieval was required for successful bungarotoxin staining, presumably due to the relatively small size of this molecule compared to monoclonal antibodies typically used for immunohistochemistry.

### Sample Imaging and Image Processing

Epifluorescence and confocal microscopy were used to evaluate AChR staining in 2D samples. Epifluorescence images were obtained by using a DM4000 B microscope (Leica) with attached Retiga 2000RV camera (QImaging, Surrey, BC, Canada), coupled with ImagePro 6.2 software (Media Cybernetics, Inc., Bethesda, MD, USA). These epifluorescence images were subsequently quantified for fluorescence events by a blinded observer. Fluorescence events were classified as occurring directly adjacent to a bead, directly underneath (“at”) a bead, or not associated with a bead. Total bead events were the sum of the “adjacent” and “at” bead fluorescence. Total fluorescence events were the sum of the “adjacent” bead fluorescence, “at bead” fluorescence, and non-bead fluorescence events.

Confocal micrographs of cells on 2D samples (see C2C12 Cell Response to Solution-Phase Agrin and Agrin-Adsorbed Microspheres on 2D Fibrin Gels *In vitro* and C2C12 Cell Response to Covalently Bound Agrin Microspheres on 2D Fibrin Gels *In vitro*) or 3D samples (see 3D *In vitro* Model System for Evaluation of the Response of Rat MDCs to Agrin-Containing Fibrin Materials Combined with Mechanical Strain) were captured on an FV10i confocal microscope (Olympus America, Center Valley, PA, USA). In both cases, DAPI staining (405 nm laser at 9.8%) was used to identify cell location by nuclear staining. Autofluorescence was used to identify microspheres (2D samples; see **Figure 4**) or fibrin scaffolds (3D samples; see **Figures 5** and **6**), and these were collected with a 473 nm laser at 16.2%. Alpha-bungarotoxin staining was collected with a 635 nm laser at 68.4%. For each channel (DAPI, autofluorescence, and alpha-bungarotoxin staining), a variable barrier function supplied by the microscope manufacturer (Olympus) was used to automatically select the bandpath wavelength range for a particular fluorescence dye. The dye sets selected for the automatic wavelength detector were DAPI (for the 405 nm laser and DAPI staining), Alexa Fluor 488 (for the 473 nm laser and fibrin autofluorescence staining) and Alexa Fluor 647 (for the 635 nm laser for alpha-bungarotoxin staining), and sensitivities were 60.4, 50.0, and 48.4% for each, respectively.

For these confocal images, regions containing cells were first identified by DAPI staining. These regions containing cells were then imaged randomly. That is, the regions of the scaffold materials were randomly selected for areas with cells. Once these random cellular areas were selected, three-dimensional projections of sequential confocal z-stacks were obtained using FIJI image processing software as described previously (Schindelin et al., 2012). Briefly, the top and bottom of the nuclei (DAPI) staining were used to estimate the top and bottom of the cells. A Z-stack image was then collected from the bottom to the top of the nuclei for each channel noted above (DAPI, beads or fibrin, and alpha-bungarotoxin). Regions containing beads were then cropped from the randomly selected cellular regions. As such, each final rendering comprises one viewing angle of a 3-D confocal z-stack projected into a 2-D image.

For the 2D samples, 2D projections of the images (see **Figures 4A,C,E,G**) were obtained by importing 3D projections of individual color channels obtained in the FIJI software to ImageJ 3D Viewer (Schmid et al., 2010) and manually adjusting each color threshold until DAPI, beads, and bungarotoxin levels were visually similar to the native 3D projection. Subsequently, the images were further processed by thresholding each to the same level for alpha-bungarotoxin and removing the DAPI (cell nuclei) signal (**Figures 4B,D,F,H**).

Acetylcholine receptor localization in 3D samples was imaged only with confocal microscopy (no epifluorescence). Methods for confocal microscopy were identical to those described above for 2D samples, except that autofluorescence was use to visualize fibrin rather than microspheres (possible due to the use of a different type of microsphere for these 3D experiments). As in the 2D samples, areas for imaging were selected by viewing DAPI signals (to select regions containing cells) before images of the α-BTX stain and fibrin autofluorescence were captured. Prior to creating a 3D projection of the collected Z-stacks, a representative region of uniform intensity in the area of fibrin autofluorescence was located from one confocal Z-slice from each sample. Intensity within each region was then quantified in each color spectrum (405 nm wavelength for DAPI, 473 nm for the fibrin scaffold, and 635 nm for α-BTX) and the mean value from the selection region’s histogram was recorded. These intensities were then averaged within each treatment group for each color (blue, green, or red channel), the treatment group with all color averages nearest the center of the possible intensity range was identified, and differences in selection intensity between each sample and the recorded mean were calculated for each color channel. To allow comparison of all samples, brightness was normalized across samples by adjusting maximum intensity in the FIJI software B&C (Brightness & Contrast) window for each color channel of each Z-stack image by the calculated difference from the mean. 3D projection was then performed in the FIJI software by using normalized Z-stacks, as described above for 2D samples. To quantify the levels of AChR clustering within the 3D samples, regions of bungarotoxin staining (as indicated by red color) were selected in Photoshop by a blinded observer. The intensity per unit area for all selected images was determined and averaged for each treatment group.

### Statistical Analysis

Where appropriate, we used a one-way analysis of variance (ANOVA) with Tukey’s *post hoc* analysis in Minitab. Probability (P) values less than 0.05 were taken to be statistically significant.

## Results

### Effect of Agrin-Adsorbed Microspheres on Cultured C2C12 Cells in 2D Fibrin Gel *In vitro*

**FIGURE 3:**
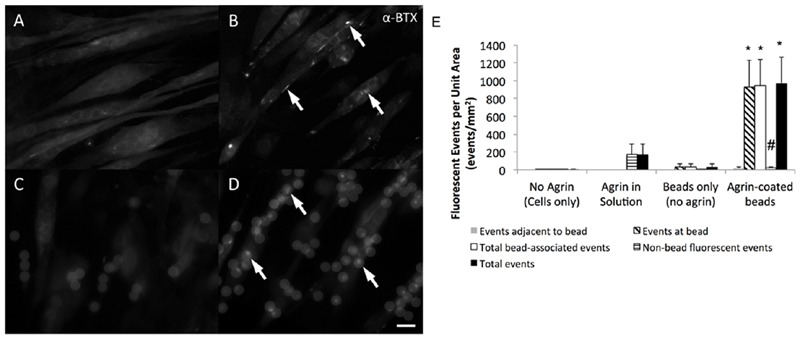
**Efficacy of agrin treatment in 2D.** Representative epifluorescent micrographs of differentiated C2C12 cells stained with fluorescent α-bungarotoxin summarize the response of cultured muscle cells to agrin presented for 16–24 h prior to staining. **(A)** Unconditioned cells are diffusely fluorescent when stained, indicating the presence of AChRs within the membrane. **(B)** When treated with agrin-conditioned culture medium, AChRs cluster together in a spatially uncoordinated fashion. **(C)** Though untreated polystyrene microspheres are visible after addition in suspension due to autofluorescence, there is no AChR clustering response. **(D)** AChR clusters are visible in the membranes of cells contacting microspheres carrying surface-adsorbed agrin in a spatially coordinated fashion. Arrows highlight examples of AChR clustering. Scale bar represents 15 μm in **(A–D)**. **(E)** Quantification of bungarotoxin fluorescence events for each of the conditions shown in **(A–D)**. Events were classified as “adjacent to the bead,” “at the bead,” or events not associated with the bead. Total bead-associated events are combination of “adjacent” and “at bead” events. Total events are combination of “adjacent,” “at bead,” and events not associated with the bead. ^∗^ Indicates significantly greater than all other groups (*P* < 0.05 by Tukey’s *post hoc* test). # indicates significantly greater than all other groups except agrin in solution (*P* < 0.05 by Tukey’s *post hoc* test). *N* = 4 from a single experiment conducted multiple times. Error bars indicate standard error of the mean.

C2C12 cells readily attached to fibrin gels and exhibited an elongated morphology during 7 days of culture in differentiation medium. As observed by fluorescent labeling with α-BTX, these elongated cells often expressed AChRs diffusely within their membranes (**Figure 3A**). However, in the absence of exogenous agrin supplementation, there was no clustering or other observable spatial organization of these receptors. In samples treated with agrin dissolved within the culture medium, detectable AChR clusters were observed on numerous cells within 16 h after treatment, though again without spatial organization or predictability (**Figure 3B**). Addition of polystyrene microspheres in the absence of agrin had no detectable effect on cellular AChR quantity or location (**Figure 3C**). In contrast, addition of agrin-adsorbed microspheres to the cultures resulted in strong AChR labeling at areas of cell-microsphere contact after as little as 16 h (**Figure 3D**). Furthermore, there was little or no detectable AChR clustering on cells distant from an agrin microsphere. The images shown are representative images of those taken from four different samples (*N* = 4) of a single experiment that was conducted multiple times. **Figure 3E** shows quantification of the number and types of fluorescence events as quantified by a blinded observer for the images shown in **Figures 3A–D**. Clearly, most of the events observed occurred at/underneath the bead, not adjacent to it by this method of quantification. Agrin in solution had a relatively large number of total events, which were non-bead events since no beads were present. However, agrin associated with the microbeads led to significantly more total events and also showed a reduction in the number of non-bead fluorescent events.

### C2C12 Cell Response to Covalently Bound Agrin Microspheres on 2D Fibrin Gels *In vitro*

**FIGURE 4:**
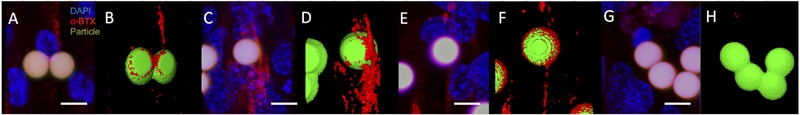
**Acetylcholine receptor clustering adjacent to agrin-presenting microspheres in 2D. (A,B)** After 16–24 h of presenting microspheres by suspension within culture medium, AChR clusters (red) are present in differentiated C2C12 cells cultured on fibrin and adjacent to microspheres (green) presenting surface-adsorbed agrin, even in complex cell topographies. **(C,D)** Similar behavior is observed after 5 days of suspension treatment in cultured cells adjacent to microsphere surfaces with covalently bound agrin. **(E,F)** This behavior is maintained when covalently bound agrin microspheres are embedded within the underlying fibrin gel for the entire 8-day period of culture. **(G,H)** Region in which cells were present but no bungarotoxin staining was observed. Negative controls (i.e., microspheres with no agrin) appeared similar to those shown in **(G,H)**. **(A,C,E,G)** Represent selected rotational views of a 3D rendered confocal image stack, while **(B,D,F,H)** represent the same 3D renders after application of a thresholding algorithm to visually reconstruct microscopic surfaces. Images are from a randomly selected area of cells. Four separate samples were used to verify repeatability (*N* = 4). Scale bar represents 10 μm.

To further evaluate the characteristics of the C2C12 cell response to microsphere-mediated agrin delivery on a 2D fibrin surface, samples with agrin-adsorbed or covalently bound agrin were imaged with confocal microscopy and reconstructed in ImageJ. The use of confocal imaging with laser-power light sources allowed us to achieve greater contrast and thus better visualize the behavior at the microsphere-cell interface. Although, confocal images showed that C2C12 cells sometimes exhibited AChR clusters (red) a small distance away from agrin-adsorbed microspheres (**Figures 4A,B**) after 16–24 h of treatment, agrin clustering was located near the beads, in agreement with the results shown in **Figure 3**.

To evaluate the impact of agrin on AChR clustering over longer time periods (from 5 to 8 days), covalently bound agrin microspheres were used. In contrast to observations with agrin-adsorbed microspheres (**Figures 4A,B**), AChR clusters near covalently coupled agrin microspheres were both larger in total size and more diffusely spread from the area of cell-microsphere contact (**Figures 4C,D**). When microspheres with covalently bound agrin were directly embedded into the underlying fibrin gel to better mimic the spatial restriction of agrin to the neuromuscular synapse that would be found *in vivo*, imaging revealed that C2C12 cells attached to the fibrin gel, elongated, and exhibited AChR clusters (**Figures 4E,F**).

We note that not all beads showed bungarotoxin staining (indicated by red), and an area of such behavior is shown in **Figures 4G,H**. Images shown are from a single experiment conducted multiple times in quadruplicate (*N* = 4). The images shown in **Figure 4** (2D experiments) are projections of the Z-stack, meaning that fluorescent events anywhere in the Z-stack can be observed.

### 3D *In vitro* Model System for Evaluation of Agrin-Containing Fibrin Materials Combined with Mechanical Strain

**FIGURE 5:**
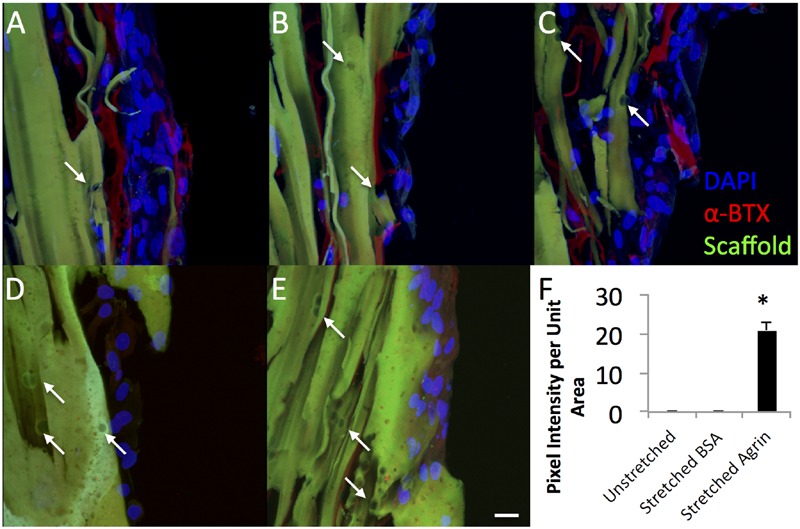
**Dramatic impact of bioreactor preconditioning on AChR expression and organization in 3D tissue engineered constructs seeded with rat MDCs. (A–C)** Bioreactor preconditioning in constructs with embedded agrin-presenting microspheres induces robust appearance large tracts of AChR clusters (red), indicating that mechanical stretch and agrin presentation may act synergistically. Very little AChR clustering and no large tracts of AChR clusters were observed for **(D)** unstretched (no bioreactor preconditioning) constructs with embedded agrin-presenting or **(E)** bioreactor preconditioned constructs with embedded BSA-presenting microspheres. Green color indicates fibrin construct (by autofluorescence) and the DAPI staining (blue) indicates an immature cell phenotype. **(F)** Shows the quantification of the bungarotoxin intensity as pixel intensity per unit area as determined by a blinded observer for three separate images (*n* = 3) from a bioreactor experiment. Error bars in **(F)** indicate standard error of the mean and ^∗^ indicates *P* < 0.05 of stretched (bioreactor preconditioned) constructs compared to unstretched (static) agrin-presenting microspheres or stretched (bioreactor preconditioned) BSA-presenting beads. Scale bar represents 20 μm.

To gain insight into the utility of this approach for *in vivo* rat models that use bioreactor pre-conditioning, we also evaluated the impact of agrin-coated microspheres on rat primary cell cultures. For these studies, rat MDCs were exposed to agrin microspheres and/or uniaxial mechanical stretch in a 3D fibrin gel (see **Figure 1**). In contrast to 2D fibrin gels seeded with C2C12 cells, representative images of rat MDC-seeded 3D fibrin biomaterials revealed little evidence of AChR labeling and no AChR clustering on statically cultured constructs. This was true whether the cultured rMDCs were in the absence or presence of covalently bound agrin microspheres (**Figures 5D,E**, respectively). Rat MDCs seeded on BSA-presenting control microspheres (i.e., no agrin) embedded in 3D fibrin scaffolds displayed diffuse AChR labeling after bioreactor preconditioning (**Figure 5C**), reminiscent of observations with C2C12 cells in the static 2D model (see **Figure 3A**) and at levels similar to those in statically cultured cells (**Figures 5D,E**). These AChRs with BSA-microspheres with bioreactor pre-conditioning were spatially unorganized, indicating that mechanical strain alone does not induce AChR clustering. Results are from a single bioreactor experiment in triplicate (*N* = 3).

**FIGURE 6:**
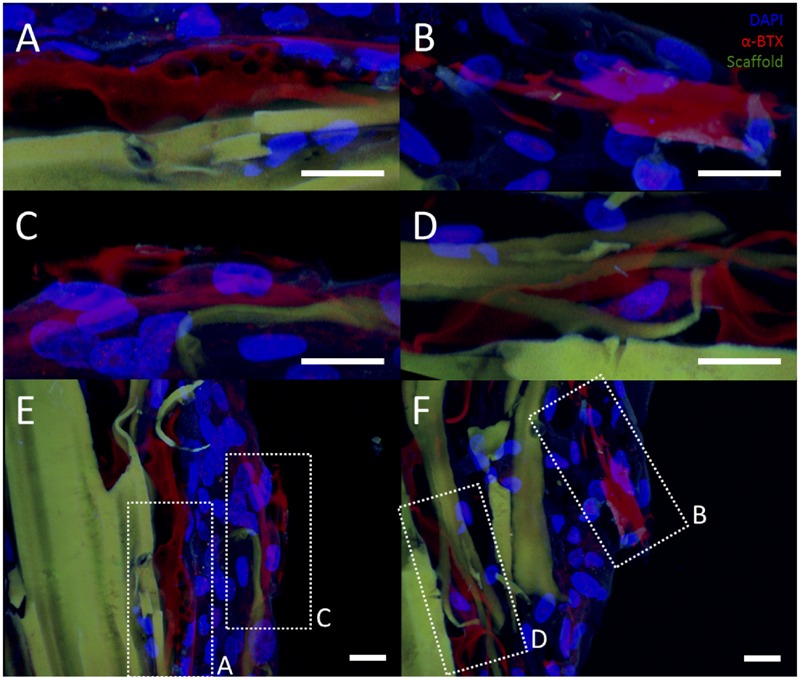
**Representative examples of AChR clustering in a bioreactor-preconditioned agrin-presenting tissue engineered construct.** Shown are rat MDCs (blue nuclei) seeded on the surface of fibrin scaffolds (green autofluorescence) carrying embedded SiO2 microspheres with covalently surface-bound agrin. Cells exhibited AChR clustering (red) after constructs were subjected to 5 days of cyclic mechanical strain. In contrast to the discrete geometry of AChR clusters observed under static culture with C2C12 cells (see **Figures 3** and **4**), AChRs in these rat MDCs cultured on bioreactor-conditioned agrin-presenting scaffolds clustered into extensive structures of complex geometry **(A–D)** more reminiscent of the appearance of native MEPs *in vivo*. By contrast, AChR clusters were never observed under static 3D culture conditions (compare to **Figure 5D**), indicating mechanical stretch and agrin presentation may act synergistically in the formation of mature MEP-like structures *in vitro*. **(A–D)** Represent selected magnified views from a 3D-projected confocal z-stack, and are shown in the context of the larger section (**E,F**, compare to **Figures 5A,C**). Scale bars represent 20 μm.

However, rMDCs seeded on agrin-presenting microspheres embedded in 3D fibrin scaffolds revealed extensive clusters of AChRs after bioreactor conditioning. **Figures 5A–C** represent three different fields of view from this treatment condition. Interestingly, these clusters were not spatially limited to discrete areas of cell-particle contact nor were they elliptical in shape as was often observed in 2D culture (compare to **Figure 3D** and **Figures 4A–F**). Instead, they featured a large, interconnected, complex geometry (**Figures 6A–D**). Representative images in **Figures 6A–D** are higher magnification images of those in **Figures 6E,F**, which are in turn higher-magnification areas from **Figures 5A,C**. The images shown in **Figures 5** and **6** are projections of the Z-stack, meaning that fluorescent events anywhere in the Z-stack can be observed.

## Discussion

We have previously described (Machingal et al., 2011; Corona et al., 2012, 2014) a technology that combined biomaterials (decellularized bladder acellular matrix), rat MDCs, and cyclic mechanical stretch in a bioreactor to create a TEMR technology for improved functional restoration of VML injury. Those studies documented that TEMR implantation into surgically created VML injuries in mouse LD (Merritt et al., 2010a; Hinds et al., 2011) and rat TA muscles succeeded in restoring 60–70% of native skeletal muscle force generation within 2 months of implantation, which was significantly greater than no repair (Machingal et al., 2011; Corona et al., 2012) scaffold alone (Machingal et al., 2011) or cells on matrix without preconditioning (Corona et al., 2012). These studies clearly demonstrated the importance of bioreactor preconditioning to the development of muscle repair constructs, but the lack of complete restoration of functional restoration indicates that additional improvements are required.

In native tissue during development, AChR are diffuse within the myotube. A motor axon nerve terminal secretes agrin to induce post-synaptic differentiation. Several additional steps lead formation of the NMJ, which can persist for the lifetime of the tissue.(Sanes and Lichtman, 2001). However, in adult muscle, the loss of the NMJ and related AChR clustering leads to muscle atrophy or muscle loss (Wang et al., 2013). Our overarching hypothesis is that one reason for the lack of complete restoration of function with tissue-engineered approaches to VML is related to the lack of innervation from the native peripheral nervous system, which is in turn related to loss of the key functional phenotype of AChR clustering. That is, we are suggesting that a key limitation to optimizing tissue engineering strategies for functional restoration of VML injuries is the lack of AChR clustering on the implanted constructs, leading to adverse phenotypic changes that diminish the potential degree of functional recovery possible, even when re-innervation of the engineered constructs is eventually achieved.

The precise role of AChR clustering is not entirely clear. For example, it is possible that areas of AChR clusters are not the location of NMJ formation (Kummer et al., 2006) though these AChR clusters may play a role in that formation given that the formation of these clusters precedes motor neuron innervation (Sanes and Lichtman, 1999, 2001). However, regardless of the precise mechanism, it is well-established that AChR clustering is an important determinant of the phenotype of muscle cells. Thus, by extension, the ability to generate and maintain clustered AChRs in tissue-engineered constructs would be expected to predispose the muscle cell phenotype to be more reminiscent of that expected of native muscle. In this scenario, one might anticipate the functional outcomes of VML repair to be comparable to that observed in smaller types of muscle injury in which NMJ do form more rapidly (Frank et al., 1975; Carlson and Faulkner, 1988; Dedkov et al., 2002).

In this report, we have described a biomaterial-based strategy for the spatially controlled delivery of neural agrin and have characterized the cellular response to these materials for both a cell line as well as with rat primary cells that would be suitable for isogeneic grafting procedures for rat *in vivo* models. We elected to use fibrin hydrogels as the biomaterial for these studies for several reasons. First, fibrin is a native component of the wound healing process and its degradation products are non-toxic. Fibrin has also been extensively studied as a tissue engineering substrate for skeletal muscle (Bach et al., 2006; Dhawan et al., 2007; Bian and Bursac, 2009; Khodabukus and Baar, 2009; Koning et al., 2009; Hinds et al., 2011; Page et al., 2011; Martin et al., 2013). In the long-term view, fibrin would be suitable for clinical applications as it can be autologously sourced (Linnes et al., 2007) from a patient’s blood, minimizing the likelihood of an immunological response. Further, fibrin degrades naturally *in vivo* on the order of weeks (Linnes et al., 2007), and can be fabricated to possess mechanical stiffness similar to native skeletal muscle [Young’s modulus: 110–320 kPa for a 3D fibrin construct used in this report (Scott et al., 2011) vs. 7–127 kPa for native muscle tissue (Levinson et al., 1995)]. A key rationale for the use of fibrin in these proof-of-concept studies, however, was that it can be fabricated by methods compatible with the immobilization of agrin-presenting microspheres within the context of a material of sufficient strength for bioreactor pre-conditioning (Scott et al., 2011). The ability to achieve immobilization of the agrin-microspheres during the fabrication of the materials is important but is not readily achieved by the bladder acellular matrix constructs used in our previous studies. Nonetheless, the approach reported here would likely be compatible with any biomaterial system in which the agrin-microspheres could be embedded as described here for fibrin.

The immobilization of agrin-presenting microspheres as reported herein is important as the spatial arrangement mimics the immobilization of agrin within the axonal basal lamina at a discrete point of contact with the adjacent muscle cell at the developing synapse (Wu et al., 2010). Although, AChR clustering has been described in response to adsorbed agrin microspheres in the literature (Daggett et al., 1996), the current report provides a much greater level of experimental detail *in vitro* of potential relevance to skeletal muscle tissue engineering *in vivo*. More specifically, we evaluated not only adsorption but also covalent linkage of agrin to microspheres, and did so within the context of an implantable biomaterial system. This biomaterial-mediated spatial delivery of agrin to muscle cells *in vitro* is one step toward testing the hypothesis that an altered skeletal muscle cell phenotype is ultimately responsible for lack of full functional recovery following implantation in tissue engineered muscle constructs for the treatment of VML injuries- as would be expected, for example, for any skeletal muscle cell exposed to long term neuronal denervation. Thus, it is important to note that the goal of these studies was not to create functional muscle tissue *in vitro*, but rather, to illustrate the ability to modulate key aspects of muscle tissue formation *in vitro* with the ultimate goal of using these initial findings to create more enabling technologies for muscle repair *in vivo*.

Specifically, after 7 days of culture in 2D fibrin gels, microspheres were added to C2C12 cells (**Figures 3C,D**). Despite microsphere autofluorescence, α-BTX staining was readily detectable. With agrin presented in solution, spatially uncoordinated AChR clusters were observed (**Figure 3B**). By contrast, agrin microspheres adjacent to cells (**Figure 3D**, see arrows) were often observed to have an adjacent fluorescence bright spot at the cell interface. Since C2C12 cells expressed some AChRs even in the absence of agrin, we conclude that the major impact of agrin is not on the presence of AChR receptor expression *per se*, but rather on the extent of AChR clustering. This is borne out in the quantification of fluorescence events in the C2C12 cells where agrin coated beads led to significant levels of fluorescence events compared to beads only (no agrin), cells not exposed to agrin, or cells exposed to solution phase agrin. It is noteworthy that the use of agrin adsorbed to beads led to a decrease non-bead fluorescence events (indicated by # in **Figure 3E**), suggesting that the localization of the agrin promotes more clustering of the AChR in cells cultured on two-dimensional materials.

3D reconstruction of confocal images revealed that agrin microspheres directly contacted cells after settling from suspension. Further, AChR clusters were closely associated with the cell-microsphere interface (**Figures 4A,B**). Although, hydrogels are known depots for protein delivery (Davis, 1974; Gombotz and Pettit, 1995; Cleland et al., 2001; Mellott et al., 2001; Hatefi and Amsden, 2002; Leach and Schmidt, 2005), diffusion-based release of the protein (Leach and Schmidt, 2005) leads to eventual depletion from the system. By contrast, coupling agrin to the surface of a microsphere allows spatial control of both location and dose, preventing diffusion and providing a biomimetic mechanism (Wu et al., 2010) by which AChR clustering can be maintained by continual presentation. The lack of diffusion away from the microsphere carrier suggests that AChR clustering could potentially be maintained throughout bioreactor pre-conditioning and even until innervation occurs in an *in vivo* implantation scenario.

In contrast to adsorbed agrin microspheres, which were only added in suspension to C2C12 cells over shorter time points, covalently bound agrin microspheres were added to C2C12 cells on 2D fibrin for the last 5 days of culture. Though, the AChR clusters observed were less spatially restricted to areas of cell-particle contact than those observed near agrin-adsorbed microspheres (compare **Figures 4C,D** to **Figures 4A,B**), they were often larger, potentially as a result of longer-term agrin presentation. These initial data demonstrate the utility of covalently coupling agrin to microspheres for longer-term signaling of muscle cells. Covalently linked agrin microspheres were also embedded within the fibrin hydrogel to create a more native-like agrin-presenting substrate for cell attachment. Confocal imaging revealed abundant AChR clusters on cell surfaces near immobilized microspheres (**Figures 4E,F**), demonstrating that immobilized functional agrin microspheres could be easily incorporated into 3D scaffolds. As discussed below for the 3D constructs, the effects of more diffuse AChR labeling remain to be elucidated, but the ability to form and/or maintain AChR clustering is an important phenotypic characteristic achieved by the approach described.

Due to their ready availability and expandability, C2C12 cells were used in the initial *in vitro* experiments to identify key parameters (e.g., agrin:microsphere ratio, number of microspheres). However, for the 3D model system, we evaluated the effects of agrin on rat MDCs, as this would be most applicable to their potential use in *in vivo* rodent models of VML injury repair and regeneration (Moon du et al., 2008; Machingal et al., 2011; Corona et al., 2012). We note that that 3D fibrin scaffolds used a sacrificial templating approach (Scott et al., 2011) able to promote the aligned phenotype of skeletal muscle. For fabrication of these 3D fibrin constructs, it is necessary to use solvent washes (in this case, acetone) to remove the sacrificial fibers. For this reason, SiO_2_ particles were used because they do not readily dissolve in acetone the way that polystyrene beads used in the 2D gels would. We do recognize that neither the polystyrene nor SiO_2_ microspheres used in these studies will ultimately be suitable for use *in vivo*. Instead, a biodegradable microsphere system that is not readily soluble in acetone would be necessary to prevent a large foreign body/inflammatory response to the agrin microspheres. However, we elected to use these materials in these proof-of-concept studies to avoid confounding factors related to carrier degradation (e.g., release of soluble agrin with degradation of the microsphere).

For these experiments, the effect of mechanical preconditioning on AChR clustering on rat MDCs in surface-seeded constructs was evaluated in combination with covalently linked agrin microspheres or with microspheres not containing agrin (i.e., in scaffolds incorporating microspheres with covalently linked BSA). Little AChR expression was observed in the statically cultured (i.e., no bioreactor preconditioning) group with agrin microspheres (**Figure 5D**). Similarly, a small amount of AChR can be observed in bioreactor preconditioned constructs incorporating BSA microspheres (**Figure 5B**); however, this labeling was faint, was not detected by the blinded observer, and was similar to that observed in differentiated C2C12 cells not treated with agrin (compare to **Figures 3A** and **3C**). In contrast, AChR clustering was exclusively observed in preconditioned, agrin-presenting constructs (**Figures 5A–C**) and was widespread across the cell-seeded surface. Combined with the randomized method of selecting areas for imaging (see Materials and Methods), these results strongly suggest that AChR clustering results from a synergistic effect of mechanical stretch and exogenous agrin supplementation. We did not investigate the precise mechanism(s) behind this behavior and this remains an area for future investigation. However, given that intracellular calcium levels play a key role in AChR clustering in response to agrin (Megeath and Fallon, 1998; Tseng et al., 2003) this may be a logical starting point for these future investigations.

An interesting finding from these studies is related to the morphology of the AChR after the bioreactor pre-conditioning. The accumulation of AChRs in the MEP of mature synapses takes on a tortuous morphology (Marques et al., 2000). AChR clusters observed in preconditioned agrin constructs (**Figures 5A–C**) were not confined only to the cell-microsphere interface as was often observed in 2D (see **Figures 3D** and **4**). Instead, AChRs clustered into more extensive structures of complex geometry (**Figure 6**, which shows **Figures 5A–C** in higher detail), reminiscent of the appearance of native MEPs *in vivo*.

Clearly, the alpha-bungarotoxin staining shown with agrin-beads in the 3D system (**Figures 5** and **6**) is quite diffuse in nature and appears to occur over multiple cells (**Figures 5A–C**). For MEP formation, only one set of clusters would be necessary, but the presence of a diffuse pattern of AChR clustering would not preclude the formation of MEP formation as the AChR clusters not involved in MEP formation could be “pruned.” Regardless, it is apparent that the combination of mechanical stretch and agrin presentation acted synergistically *in vitro*, though the effects in an *in vivo* model remain to be elucidated.

There are several limitations in this report that warrant further study. First, the microsphere system delivered only agrin, though other molecules such as neuregulin likely play a role (Kummer et al., 2006). Thus, exploration of other molecules alone or in conjunction with agrin may be able to achieve even more relevant cell-level effects. Second, we also note that no effort was made to optimize the levels of agrin on the microsphere surface, which could impact AChR clustering. In addition, for the studies reported herein, we simply delivered a sufficient number of agrin-coated microspheres to elicit a response (i.e., AChR clustering). It may be necessary to titrate the number of microspheres per cell and ultimately achieve a ratio of approximately 1 microsphere per cell in order to have a single set of AChR clusters on the cell in a manner similar to the developmental process. In this regard, agrin is known to achieve clustering of existing AChR. However, it is possible that the use of agrin could lead to long-term modifications in the mRNA or protein expression profiles for cells. These could be investigated by quantitative reverse-transcriptase polymerase chain reaction (qRT-PCR) or Western blotting, respectively. However, the use of immunohistochemical imaging used in our studies is preferred for identifying the spatial localization the existing AChR and was the rationale for this approach to visualization and quantification of our data. Finally, we did not document the function of the AChR clusters so achieved, but rather demonstrated the conditions required for the *in vitro* establishment and morphology of the AChRs. While the conditions to achieve establishment and morphology of the AChR clusters are clearly important prerequisites in the technology development pathway for improved repair of VML injuries, future studies will need to assess the functional outcomes, likely with *in vivo* studies.

In short, the results described in this report critically expand upon existing studies from the literature documenting the potential utility of agrin supplementation to improved skeletal muscle tissue engineering approaches for VML injury repair (Bian and Bursac, 2012; Ko et al., 2013). Primarily, all studies of this nature to date have evaluated the effects of *soluble* agrin stimulation of muscle constructs, either *in vitro* alone (Bian and Bursac, 2012) or *in vitro* prior to implantation *in vivo* (Ko et al., 2013). These previous studies demonstrated increased force production *in vitro* by engineered constructs (Bian and Bursac, 2012) and, potentially, accelerated innervation of constructs implanted *in vivo* (Ko et al., 2013) following agrin stimulation. The current study documents a method by which the agrin signal for maintaining AChR clusters may be maintained over longer periods of time, and moreover, may be more physiologically relevant, as the agrin presentation in our method is constrained to the region of contact, and furthermore, amenable to controlled stoichiometry to achieve and required/desired number of MEP per myotube/myofiber. Others have used co-culture approaches that allow nerve axons to achieve AChR clustering (Larkin et al., 2006; Martin et al., 2015), but such an approach could lead to competition for the endplate *in vivo*. Our approach achieves AChR clustering without competition for innervation between an existing nerve and a host nerve in an *in vivo* scenario. Although our study used fibrin hydrogels and non-degradable micro-particles, the fundamental methods would be more widely applicable to other biomaterial systems (hydrogels or other matrices such as bladder acellular matrix or small intestinal submucosa) and degradable microparticle systems. That is, this approach may be used in combination with other strategies being investigated that use other materials, cells sources, or pharmacological approaches. As such, these studies represent a potentially useful extension to previous tissue engineering approaches to skeletal muscle regeneration and also provide a bridge to more enabling technologies to existing TEMR technology platforms for treatment of VML injury.

## Author Contributions

Conception, design, data collection and interpretation, and drafting of manuscript (JBS). Conception and design of experimental work, analysis of data, revising/editing manuscript (CW, BC, BH). Design of experimental work, analysis and review of data, revising/editing manuscript (MD). Conception, design, interpretation of data, drafting and revising/editing manuscript (JMS, GC). All authors are accountable for all aspects of the work.

## Conflict of Interest Statement

JBS, CW, BC, BH, JMS, and GC have a pending patent application related to this work. Patent application number is US14348273.

The reviewer CP declares a shared affiliation and previous co-publications with the authors BH and GC. While Frontiers can attest to the objectivity and thoroughness of the review, the Associate Editor Dr. Yuan ensured three reviewers were assigned to ensure a comprehensive and thorough review process.

## Abbreviations

α-BTX: alpha bungarotoxin
AChR: acetylcholine receptor
B&C: brightness and contrast
BSA: bovine serum albumin
CA: cellulose acetate
CaCl_2_: calcium chloride
LD: latissimus dorsi
MDC: muscle-derived cell
MEP: motor endplate
MES: 2-(*N*-morpholino)ethane sulfonic acid
NHS: *N*-hydroxysulfosuccinimide sodium salt
NMJ: neuromuscular junction
PBS: phosphate-buffered saline
rMDC: rat muscle-derived cell
siO_2_: silicon dioxide
TA: tibialis anterior
TBST: Tris-buffered saline with Tween
TEMR: tissue-engineered muscle repair
VML: volumetric muscle loss
